# The comparison of clinical outcomes between volar locking plate fixation and dual-plate fixation in high-energy distal radius fractures

**DOI:** 10.3389/fsurg.2025.1552764

**Published:** 2025-06-19

**Authors:** Lingyao Zhou, Yuling Gao, Dong Wang, Yanrui Zhao, Tianchao Lu, Hanzhou Wang, Binzhi Zhao, Jianwei Guo, Ziyi Li, Yang Liu, Junlin Zhou

**Affiliations:** ^1^ Department of Emergency Surgery, Beijing Changping Hospital, Bejing, China; ^2^ Department of Orthopaedics, Beijing Chaoyang Hospital, Capital Medical University, Bejing, China

**Keywords:** articular, specific fragment, volar locking plate, dorsal plate, radial column, distal radius fracture (DRF)

## Abstract

**Purposes:**

The aim of this study was to compare clinical outcomes of dual-plate fixation and volar locking plate fixation in high-energy intra-articular distal radius fractures.

**Methods:**

In this study, 168 patients with high-energy intra-articular distal radius fractures with either volar fragments, dorsal fragments, or radial styloid fragments, who were treated with one of three kinds of fixation methods (volar locking plate fixation, volar + radial plating fixation, and dorsal + radial plating fixation) were retrospectively reviewed and divided into three groups. Functional evaluations included DASH score, Mayo wrist score, wrist joint ROM, and grip strength percentage relative to the healthy side. Radiographic parameters assessed were volar tilt, radial inclination, radial height, and ulnar gap/step distance. Any complications were documented.

**Results:**

Significant differences were found in volar tilt, radial inclination, and gap/step distance 1 month postoperatively between the volar locking plate fixation group, the volar + radial plating group, and the dorsal + radial plating group. And the volar + radial plating group showed significant difference from the dorsal + radial plating group for the gap/step distance 1 month postoperatively (*p* < 0.01). Forty-six complications were recorded for 15 cases of the volar locking plate fixation group, 16 cases of the volar + radial plating group, and 15 cases of the dorsal + radial plating group.

**Conclusions:**

For high-energy distal radius fractures, volar locking plate fixation and dual-plate fixation achieved similarly positive functional and radiological outcomes. The dual-plate fixation method has the advantage of achieving better reduction quality.

## Introduction

High-energy distal radius fractures (DRFs) are often associated with comminuted intra-articular fractures in several planes and central impaction (1). Rikli et al. divided the distal radius into three biomechanical column structure and stated that it is necessary to undertake surgical treatment to restore articular congruity and carpal alignment. This is because the middle column, formed by the lunate fossa, serves as the main load-bearing structure and 50% of the axial load of the wrist is transferred through the radial column (2). Other studies have shown that the comminuted fracture zone is primarily located in the central region of the distal radiocarpal articular surface, with the fracture lines predominantly concentrated in the middle 1/3 and dorsal 1/3 regions (3, 4). Medoff and Kopylov further divided the intermediate column into several fragments (volar rim fragment, dorsal ulnar corner fragment, dorsal wall fragment, and free intraarticular fragment) and introduced the fragment-specific fixation concept (5, 6).

In conjunction with the recent increase in surgical fixation of DRFs, there has been a noticeable increase in the preference for volar locking plate (VLP) fixation. But VLP fixation provides insufficient ability to repair dorsal side fragments. The combined plating fixation method is an effective treatment for managing complex intra-articular DRFs. Several studies have compared the clinical outcomes of VLP and combined plating fixation in AO Type C distal radius fractures and showed similar outcomes other than in higher rates of implant removal and a compromised range of wrist motion in the combined plating fixation (7–9). The dual-plate fixation method has been introduced for the fixation of volar rim fragments and dorsal side fragments (10, 11). To the best of our knowledge, there is no consensus on the combined plate placement and clinical outcomes between different combined plate fixation methods. This study examined the dual-plate fixation methods including VLP + radial plating fixation and dorsal plating + radial plating fixation. The aim of this study was to compare clinical outcomes of the dual-plate fixation and VLP fixation methods on high-energy intra-articular distal radius fractures.

## Patients and methods

This study was approved by the Ethics Committee of Beijing Chaoyang Hospital, Capital Medical University, and the imaging material and clinical data adhered to the ethical standards outlined in the 1964 Declaration of Helsinki.

### Study design

From March 2017 to March 2024, patients diagnosed with DRFs were retrospectively reviewed in the hospital. The inclusion criterion included (1) high-energy DRFs with volar rim fragment, dorsal wall fragment, dorsal ulnar corner (DUC) fragment, or the radial styloid fragment; (2) aged ≥18 years or older; (3) fresh fractures within 3 weeks after injury; and (4) treated with either VLP fixation or dual-plate fixation including the VLP + radial plating fixation (V + R plating fixation) or the dorsal plating + radial plating fixation (D + R plating fixation). The exclusion criteria included (1) age <18 years, (2) pathological fractures, (3) noncompliance with postoperative follow-up among patients, (4) or previous disease on the affected hand.

The sample size for the current study was determined based on the reference values utilized in comparable studies considering the primary endpoint of range of wrist motion and postoperative radiograph parameters. The current study required a minimum of 56 participants in each group, based on the mean sample size calculation using two sample T-tests allowing unequal variance with significance level (*α*) of 0.05, power (1 − *β*) of 90%, with (0.2 ± 5.5 vs. 3.2 ± 2.9), (21.7 ± 4.1 vs. 19.4 ± 4.3), and (51.66 ± 8.90 vs. 58.75 ± 5.90) for each method respectively based on previous studies (9, 12).

**Figure 1:**
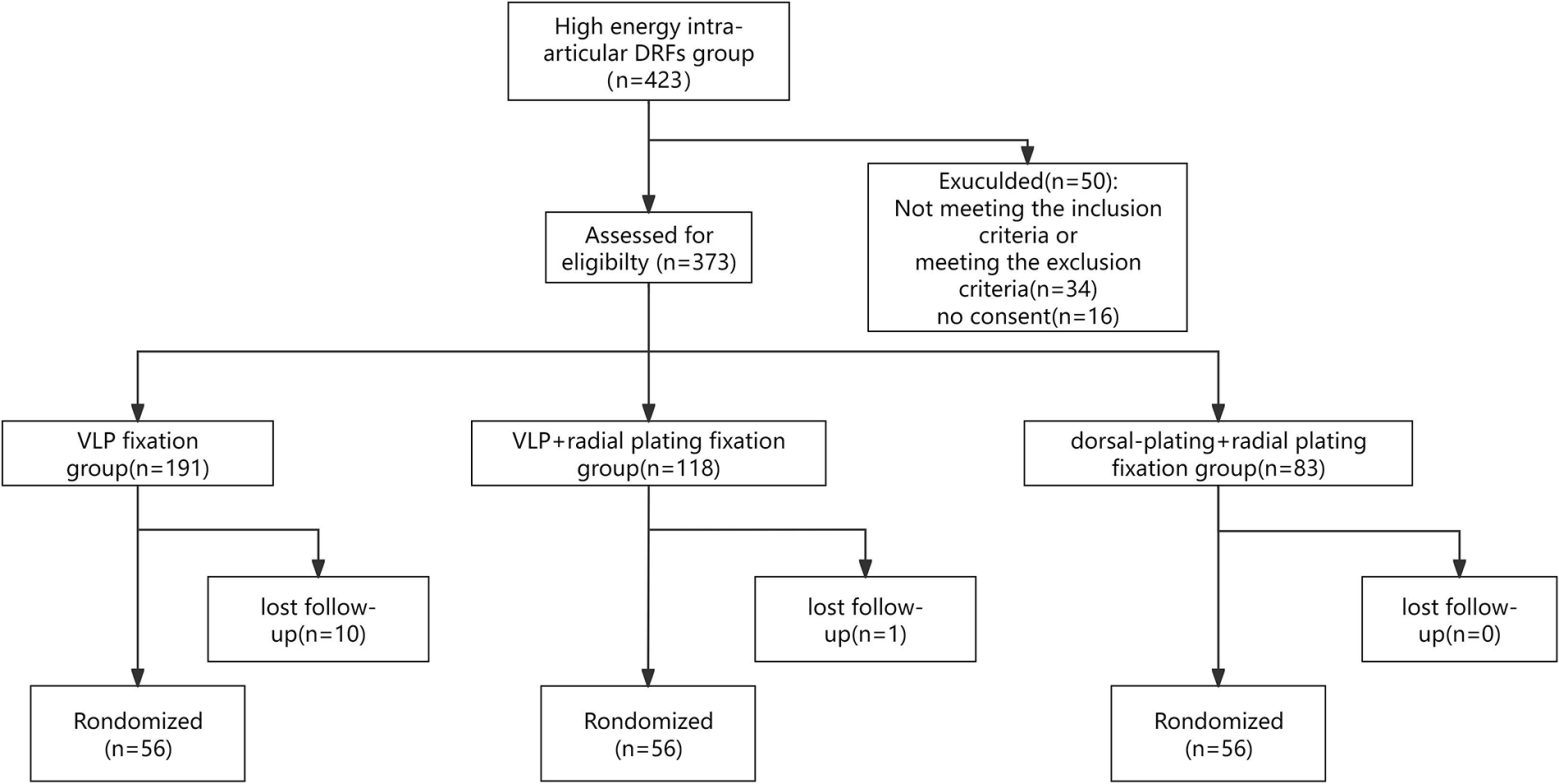
The flowchart of patient selection.

The process of retrospectively selecting patients can be seen in Figure 1. To minimize bias, patients were randomly selected for analysis from the subgroups. Each group contained patients treated with volar plate fixation and patients treated with dual-plate fixation. The patients with high-energy DRFs were treated with volar plate fixation during the years of 2017 to 2024 and dual-plate fixation during the years of 2021 to 2024. All patients were informed of the treatment choice and plan. After obtaining consent, the treatment commenced. Each patient was numbered and randomly selected from the 56 patients for each group.

### Surgical technique

The surgical procedure was conducted with the patient under general anesthesia, along with a brachial plexus block and tourniquet. For the VLP plating fixation + radial plating group, an incision was made on the palm side to access the ulnar part of the lower end of the radius bone. Through this same incision, the carpal tunnel was opened up. The area between the finger flexors towards the ulnar side and both the median nerve and thumb flexor towards the radial side allowed surgeons to expose and work on the fracture sites. The radial column was reduced through a K-wire obliquely inserted from the radial styloid to the radial shaft. And then the volar side fragments were reduced and temporarily fixed with small K-wires. If further exposure of radial column was needed, the distal insertion of the brachioradialis tendon would be released. Subsequently, placement of a volar plate followed by repair of pronator quadratus using absorbable sutures followed.

**Figure 2:**
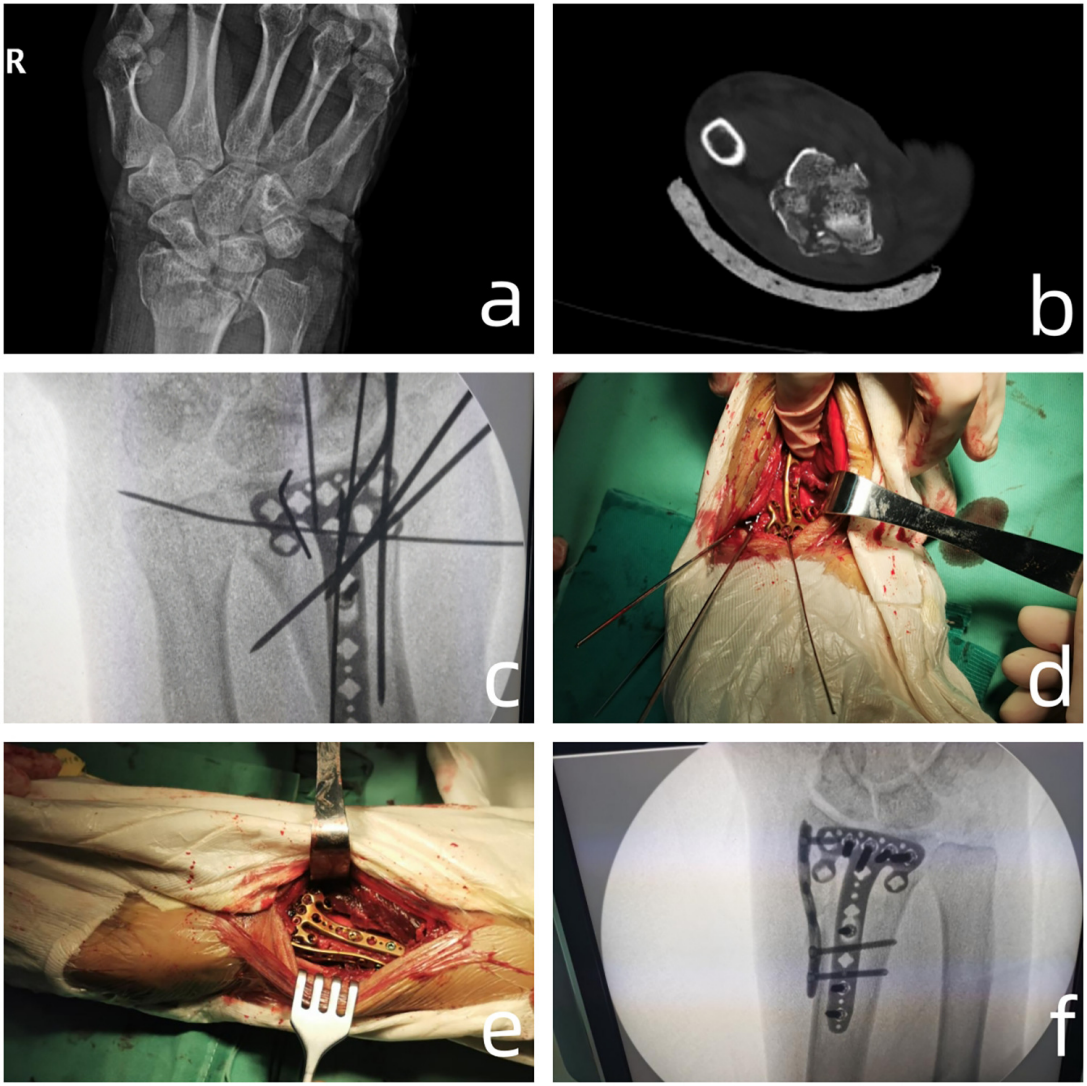
**(a,b)** The articular surface fracture as shown by x-ray and CT axial scan; **(c,d)** placement of the plate after temporary fixation with kirschner wires revealed poor reduction of the radial styloid process, leading to placement of a radial plate; **(e,f)** surgical and radiographic outcomes of dual-plate fixatio.

We used either single volar plate fixation or double-plate fixation. The volar plates used were the LCP distal radius plate ensuring each column was fixed with at least two screws. The radial plate was positioned as close to the volar aspect as possible in order to decrease impingement of the first dorsal compartment tendons. One or two bicortical non-locking (2.7 mm) screws were also placed proximally, approximating the plate to the radial column in buttress mode. The VLP plating fixation + radial plating fixation procedure can be seen in Figure 2.

**Figure 3:**
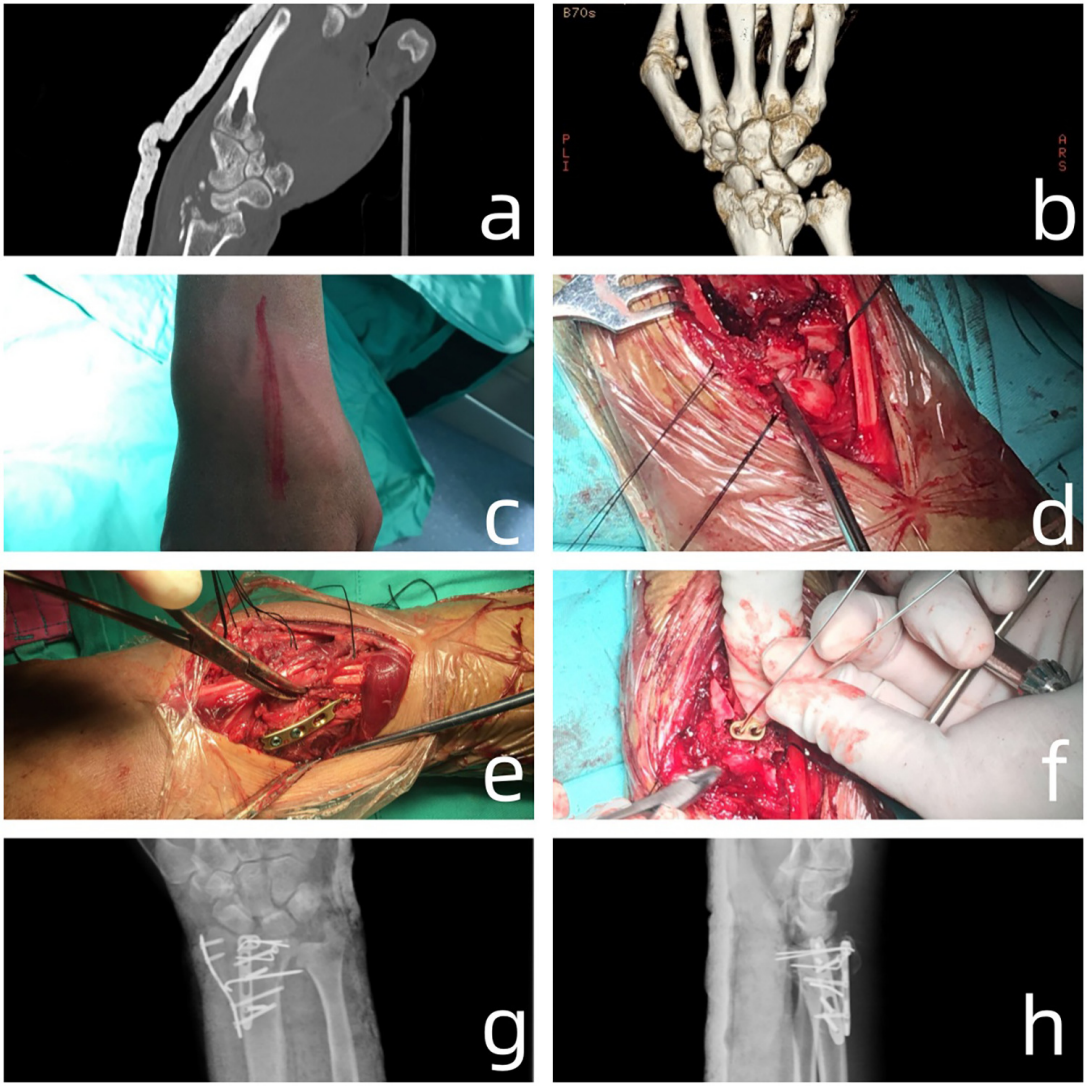
**(a,b)** Preoperative CT scan and three-dimensional reconstruction of the distal radius fracture showed that the dorsal articular surface collapsed; **(c,d)** an incision was made on the dorsal midline of the wrist, measuring approximately 7 cm in length. The subcutaneous tissue and deep fascia were incised, and the tendon sheath of the extensor pollicis longus muscle was cut. The muscle was then retracted, revealing the posterior aspect of the radius. A comminuted fracture of the distal extremity of the radius was visible, with fragmented joint surfaces and numerous small loose bone fragments; **(e,f)** A suitable L-shaped titanium plate was placed on the medial aspect of the radius, and it was fixed with several screws. A suitable radial plate was placed on the lateral aspect of the radius and fixed with several screws; **(g,h)** Postoperative radiographs of the lateral and posteroanterior views of the patient.

For the dorsal plating + radial plating fixation, an incision was made on the dorsal midline of the wrist, measuring approximately 7 cm in length. The subcutaneous tissue and deep fascia were incised, and the tendon sheath of the extensor pollicis longus muscle was cut. The muscle was then retracted, revealing the posterior aspect of the radius. A comminuted fracture of the distal extremity of the radius was visible, with fragmented joint surfaces and numerous small loose bone fragments. The lunate cartilage was also fragmented and detached. The radial styloid process was fractured and had displaced to the dorsolateral aspect. The blood clot was removed from the fracture site, and the fracture was reduced and fixed with several Kirschner wires. On C-arm fluoroscopy, the fracture reduction was deemed satisfactory. A suitable L-shaped titanium plate was placed on the medial aspect of the radius, and it was fixed with several screws. A suitable radial plate was placed on the lateral aspect of the radius and fixed with several screws. The dorsal plating + radial plating fixation procedure can be seen in Figure 3.

### Postoperative assessment

The radiographic evaluation involved obtaining relevant measurements of volar tilt, radial inclination, postoperative gap or step, and radial height at 1 month, 3 months, and 12 months postoperatively using standard immediate postoperative posteroanterior and lateral radiographs. For functional evaluation, Disabilities of the Arm, Shoulder, and Hand (DASH) score, Mayo wrist score, range of motion (ROM) of the wrist joint, and percentage of grip strength relative to the healthy wrist at 1 month, 3 months, and 12 months follow-up were evaluated and recorded. The occurrences of complications were documented during each follow-up period, including the reduction loss, infection, nerve or tendon injury, irritation related to internal fixation, complex regional pain syndrome, and implant removal.

### Statistical analysis

Statistical analysis was performed using SPSS version 22.0 (SPSS Inc, Chicago, Illinois, USA). The sample size calculation was performed by PASS version 15.0 (NCSS Inc, Kaysville, Utah, USA). Continuous variables data were analyzed using the Shapiro–Wilk (SW) test for sample sizes ≤5,000 and a run test to assess normal distribution. An independent sample non-parametric test or independent sample t test and Kendall's concordance coefficient were used according to whether the distribution was normal. Chi-square test was used for dichotomous variables. A one-way or two-way analysis of variance (ANOVA) was conducted to examine the differences in the assessments among multiple independent groups. For repeated measurements, generalized estimating equations (GEE) were conducted to analyze two fixation methods (VLP fixation and Dual-plate fixation). A *p*-value < 0.05 was considered statistically significant.

## Results

The baseline information of the two patient groups can be seen in Table 1. There were no significant differences between the three fixation methods for the high-energy intra-articular DRFs.

**Table 1:** Patients demographic data in the high energy intra-articular DRFs group.

Characteristics	VLP group (*n* = 56)	VLP + radial plating fixation group (*n* = 56)	Dorsal plating + radial plating fixation (*n* = 56)	*p*
Age, years old	43.5 ± 13.2	46.9 ± 20.7	42.5 ± 19.2	0.24
Gender (male: female), *n*	31:25	29:27	17:39	0.47
Injured side (left: right), *n*	11:45	16:40	13:43	0.31
AO classification, *n*				
(B:C)	3:53	0:56	0:56	0.19
Follow up, Month	15.3 ± 2.8	17.6 ± 2.4	15.9 ± 3.8	0.53

As shown in Table 2, significant differences in volar tilt at 1 month postoperatively were found between the VLP group (4.63 ± 9.20°), the V + R plating group (5.34 ± 10.1°) (*p* = 0.02), and the D + R plating group (5.29 ± 8.1°) (*p* = 0.01). The GEE for volar tilt was conducted and showed significant differences (*p* < 0.01, 95% Wald CI: 0.05–4.27) between the three groups.

**Table 2:** The treatment outcome in the high-energy intra-articular DRFs between three groups.

Postoperative assessments	1-month postoperative			3-month postoperative			12-month postoperative		
	VLP group	V + R plating group	D + R plating group	VLP group	V + R plating group	D + R plating group	VLP group	V + R plating group	D + R plating group
Postoperative radiograph parameter									
Volar tilt, °	4.63 ± 9.20^a^	5.34 ± 10.1	5.29 ± 8.1	6.17 ± 8.89	5.73 ± 9.84	6.27 ± 8.73	6.90 ± 7.50	6.1 ± 11.2	7.19 ± 9.28
Radial inclination, °	18.72 ± 5.12^b^	21.42 ± 3.97	20.54 ± 4.28	20.67 ± 3.23	21.67 ± 3.47	20.87 ± 2.54	20.74 ± 3.96	22.61 ± 3.81	21.92 ± 1.27
Radial height, mm	11.83 ± 1.69	10.65 ± 2.64	10.29 ± 1.72	11.69 ± 2.54	12.51 ± 1.53	11.29 ± 1.98	12.06 ± 1.69	11.97 ± 2.13	12.68 ± 0.79
Gap/step, mm	2.10 ± 1.95^c^	1.14 ± 0.63	0.74 ± 0.08^d^	0	0	0	0	0	0
Range of motion									
Flexion, °	43.62 ± 10.30	44.95 ± 7.18	41.79 ± 8.59	69.04 ± 6.21	68.26 ± 5.39	66.43 ± 13.78	76.57 ± 4.32	74.62 ± 2.53	72.76 ± 8.44
Extension, °	29.18 ± 7.34	31.36 ± 8.62	29.86 ± 9.53	49.28 ± 9.30	46.51 ± 7.63	41.38 ± 3.91	64.89 ± 7.26	62.25 ± 8.95	56.29 ± 10.86
Supination,°	72.43 ± 5.43	73.45 ± 2.63	69.29 ± 7.84	78.36 ± 8.64	76.18 ± 4.95	78.23 ± 11.43	84.38 ± 7.52	86.72 ± 5.46	86.40 ± 7.84
Pronation,°	70.94 ± 6.85	71.27 ± 5.45	73.56 ± 12.53	72.04 ± 9.65	68.61 ± 8.59	78.99 ± 7.47	81.65 ± 6.89	84.26 ± 7.03	82.53 ± 3.97
Functional assessment									
DASH score	40.09 ± 8.61	37.52 ± 11.08	39.56 ± 9.24	13.20 ± 4.96	10.95 ± 5.26	8.25 ± 6.54	3.23 ± 1.14	2.96 ± 0.19	2.89 ± 2.07
MAYO wrist score	30.29 ± 11.65	32.67 ± 9.74	30.12 ± 5.63	70.63 ± 15.03	73.47 ± 13.79	80.06 ± 9.35	88.36 ± 10.23	86.19 ± 14.07	83.29 ± 7.82
Grip strength, %	38.43 ± 10.69	40.73 ± 15.49	36.54 ± 9.60	59.36 ± 7.50	60.43 ± 3.29	59.67 ± 2.87	91.67 ± 4.92	89.76 ± 9.68	80.19 ± 10.50

Data was expressed as mean ± standard deviation or median (minimum, maximum).

For the comparison of *p* < 0.05, it was marked as a letter label, while for the other comparison, the *p* value was over 0.05.

^a^ There were significant differences in volar tilt 1 month postoperatively between the VLP and the V + R plating group (*p* = 0.02) as well as the D + R plating group (*p* = 0.01).

^b^ There were significant differences in radial inclination 1 month postoperatively between the VLP group, the V + R plating group (*p* = 0.01), and the D + R plating group (*p* = 0.01).

^c^ There were significant differences in gap/step 1 month postoperatively between the VLP group and V + R plating group (*p* = 0.01) as well as the D + R plating group (*p* = 0.03).

^d^ There was significant difference in gap/step 1 month postoperatively between the D + R plating group and V + R plating group (*p* < 0.01).

There were significant differences between the VLP group (18.72 ± 5.12°) and the V + R plating group (21.42 ± 3.97°) (*p* = 0.01) as well as the D + R plating group (20.54 ± 4.28°) (*p* = 0.01) for radial inclination at 1 month postoperatively. The GEE for radial inclination was conducted and was significantly different (*p* = 0.01, 95% Wald CI: 0.29–2.82) between the three groups.

There were significant differences between the VLP group (2.10 ± 1.95 mm), the V + R plating group (1.14 ± 0.63 mm) (*p* = 0.01), and the D + R plating group (0.74 ± 0.08 mm) (*p* = 0.03) for the gap/step distance at 1 month postoperatively. The V + R plating group showed significant difference to the D + R plating group for the gap/step distance at 1 month postoperatively (*p* < 0.01). The GEE for the gap/step was conducted and showed a significant difference (*p* < 0.001, 95% Wald CI: 0.15–3.61) between the VLP and dual-plate groups.

As shown in Table 3, the VLP group had 15 cases of complications: 3 cases of reduction loss, 4 cases of implant irritations, and 8 cases of implant removal. The V + R plating group had 16 cases of complications: 1 case of reduction loss, 5 cases of implant irritation, and 10 cases of implant removal. The D + R plating group had 15 complications: 1 case of reduction loss, 4 cases of implant irritation, and 10 cases of implant removal.

**Table 3:** Complications of treatment in the three groups.

Complication events	VLP group	V + R plating group	D + R plating group
Infection, *n*	0	0	0
Reduction loss, *n*	3	1	1
Nerve injury, *n*	0	0	0
Tendon injury, *n*	0	0	0
Implant irritation, *n*	2	5	4
Implant removal, *n*	8	10	13
Complex regional pain syndrome, *n*	0	0	0

## Discussion

Our study compared treatment outcomes of VLP fixation and two kinds of dual-plate fixation for high-energy DRFs. The results demonstrated comparable functional and radiological outcomes between the three fixation methods at the final follow-up. It is worth mentioning that the dual-plate fixation method showed better reduction ability than the VLP fixation at 1-month postoperatively. The dual-plate fixation method could be applied as an effective treatment for unstable or nonreducible DRFs. The treatment of high-energy DRFs is highly dependent on the fracture morphology situation, so the intra-articular fragments are the main deciding factor for treatment choice. It was discovered that the presence of ligament insertions could lead to recurring fracture lines, especially in many two-part fractures; thus, the term “osteoligamentary unit” was introduced (13, 14). Brink and Rikli divided the distal radius articular into four pillar parts, terming the fragment causing the shift of the carpus as the “key fragment” and presenting the four-corner concept (15). According to previous studies and our experience, dorsal side fragments are formed by dorsal wall fragments and dorsal ulnar corner (DUC) fragments. The most typical dorsal side comminuted fracture is the die-punch fracture, with lunate fossa fragment separated dorsally or impacted. The volar side fragments were predominantly localized to the intermediate column and radial column, but there is currently no standardized nomenclature for each individual fracture fragment. The volar side comminuted fractures and radial column comminuted fractures are mainly concentrated on the radial and volar zone (3). Our study compared the clinical outcomes between dual-plate fixation and volar plate fixation for different high-energy DRFs and found that the dual-plate fixation has the advantage of achieving anatomical reduction in some fracture patterns.

The volar locking plate is the preferred option due to its superior stability in resisting shear forces as a buttress. Column fixation theories can also be applied to fractures in other extremities, including tibial pilon fractures and elbow instability injuries, advocating for appropriate fixation and stabilization of each of the three columns (16, 17). The traditional plate method poses a risk of postoperative displacement due to its flat and contoured design not conforming to the volar side outline of the distal radius. In contrast, the VLP offers the advantage of a fixed-angle locking plate, providing support for both the radial and volar lunate facets (18). However, due to concern over the limited purchase opportunities for comminuted radial column fragments and the inability to prevent brachioradialis tendon pulling force, the radial column plate combined with volar locking plate fixation was proposed (19). Currently, there is little debate over the radial column plate. Galle SE conducted a retrospective review and indicated minimal complications along with objective scores consistent with restoration of normal function for radial column plating (20). Our study suggested the V + R plating fixation could achieve better recovery of the radial inclination and fracture gap/step distance at 1-month postoperatively than the single volar locking plate fixation, with similar results in postoperative clinical outcomes. Noticeably, three cases of reduction loss were found in the VLP group. Lucke-Wold BP et al. reported a case of an older patient with a re-fracture of the distal radius due to trauma and advised on the risk factors associated with older patients, including osteoporosis, obesity, and muscle weakness (21). Similarly, the reduction loss cases in this study were found to be elder patients. The tensile strength of the volar locking plate is formed by its material characteristics, plate morphological design, and biomechanical environment (22, 23). Considering that high-energy DRFs are often accompanied by die-punch fragments with the depression of the articular surface, bone growth substitutes such as BMP or putty could be used to recover the articular surface and amend plate tensile strength (24).

Though the VLP fixation could achieve satisfying outcomes for AO C3-type fractures, the choice of the dorsal plate fixation method is because the volar locking plate may not be sufficient to fully secure the dorsal side fragments (9). The die-punch fractures specifically could not be reduced by ligamentotaxis and required direct visualization for reduction. In this study, the fractures gap/step distance were mainly caused by the free dorsal fragments in the VLP fixation. This study also compared the clinical outcomes between different dual-plate fixation methods in high-energy intra-articular DRFs, which has been seldom mentioned in other studies. For the current study, the V + R plating fixation showed comparable clinical outcomes to the D + R plating fixation, except the D + R plating fixation showed better dorsal fragment reduction ability. The fragment-specific fixation concept has been widely used in the treatment the high-energy DRFs. Considering the less invasive principle, we used the dual-plate fixation through one approach rather than the combined approach. The radial column could be exposed when the tendons of the first compartment were released from their compartments at the proximal end of the tendon transition zone. The approach to the intermediate column involves retraction of the extensor pollicis longus tendon from its fascial compartment. Subsequently, the third compartment was opened in a proximal-to-distal direction along the tendon sheath, creating an ulnar-directed V-shaped incision while elevating the second compartment to expose the intermediate column. Landgren M conducted a randomized controlled study and showed that more complications, such as transient radial neurapraxia and tendon injuries or irritation, were recorded in the fragment-specific group (25). However, our study did not reveal significant complication rates for the three fixation groups. This could be attributed to the use of lower-profile plates recessed into the plate and the advancements made in surgical technique using extensor retinaculum flaps to cover the plate and minimize irritation of the extensor tendon. Additionally, our study showed that the dual-plate fixation method showed relatively less distance in the fracture gap or step after the operation than the VLP fixation method. The better reduction in dorsal side fragments could also decrease the risk of tension irritation. It is also notable that the dorsal plate fixation method showed less range of wrist flexion and extension than the VLP fixation method, though no significant differences were found. Similarly, other studies also showed restricted wrist motion in the dorsal plating group (7, 8, 26). This could be due to the room needed for dorsal plating, longer periods of immobilization, patients' original factors, or the extent of the displacement.

There are limitations to our study. Our study did not include an analysis of late complications, such as traumatic arthritis, which affects the quality of the reduction. Particularly for intra-articular fractures, subtle differences in reduction quality (for example, the dual-plate group's better restoration of articular surface) could translate into differences in arthritis rates beyond one year. Secondly, in the current study, the dorsal dual-plate fixation method seems to allow for a smaller range of extension/flexion compared to the VLP fixation method, although both achieved similar functional scores, so this needs to be further debated.

## Conclusion

For high-energy distal radius fractures, the volar locking plate fixation and dual-plate fixation methods can achieve similarly positive functional and radiological outcomes. The dual-plate fixation method has the advantage of achieving better reduction quality.

## Data Availability

The original contributions presented in the study are included in the article/Supplementary Material; further inquiries can be directed to the corresponding authors.
